# A Policy Analysis of the implementation of a Reproductive Health Vouchers Program in Kenya

**DOI:** 10.1186/1471-2458-12-540

**Published:** 2012-07-23

**Authors:** Timothy Abuya, Rebecca Njuki, Charlotte E Warren, Jerry Okal, Francis Obare, Lucy Kanya, Ian Askew, Ben Bellows

**Affiliations:** 1 Population Council Nairobi, Nairobi, Kenya

**Keywords:** Output-based approach, Reproductive health, Vouchers, Maternal health, Safe motherhood, Family planning, Policy analysis

## Abstract

**Background:**

Innovative financing strategies such as those that integrate supply and demand elements like the output-based approach (OBA) have been implemented to reduce financial barriers to maternal health services. The Kenyan government with support from the German Development Bank (KfW) implemented an OBA voucher program to subsidize priority reproductive health services. Little evidence exists on the experience of implementing such programs in different settings. We describe the implementation process of the Kenyan OBA program and draw implications for scale up.

**Methods:**

Policy analysis using document review and qualitative data from 10 in-depth interviews with facility in-charges and 18 with service providers from the contracted facilities, local administration, health and field managers in Kitui, Kiambu and Kisumu districts as well as Korogocho and Viwandani slums in Nairobi.

**Results:**

The OBA implementation process was designed in phases providing an opportunity for learning and adapting the lessons to local settings; the design consisted of five components: a defined benefit package, contracting and quality assurance; marketing and distribution of vouchers and claims processing and reimbursement. Key implementation challenges included limited feedback to providers on the outcomes of quality assurance and accreditation and budgetary constraints that limited effective marketing leading to inadequate information to clients on the benefit package. Claims processing and reimbursement was sophisticated but required adherence to time consuming procedures and in some cases private providers complained of low reimbursement rates for services provided.

**Conclusions:**

OBA voucher schemes can be implemented successfully in similar settings. For effective scale up, strong partnership will be required between the public and private entities. The government’s role is key and should include provision of adequate funding, stewardship and looking for opportunities to utilize existing platforms to scale up such strategies.

## Background

The direct costs of maternal health care are prohibitive to many women in low income countries. Fear of high costs and potential catastrophic expenditure that push a household further into poverty causes many women and their families to risk giving birth at home or delay seeking care. In these settings between one and five percent of total annual household expenditure is spent on maternal health care, rising to between five and 34 percent in case of obstetric complications [1]. Innovative approaches have been implemented to reduce the financial barriers to maternal health services in low income countries [2]. These approaches include demand-side consumer-led initiatives like cash transfers and tax rebates as well as supply-side provider-led initiatives like referral vouchers and strategies that integrate supply and demand elements like the output-based approach (OBA) [3].

In principle, OBA improves efficiency in service delivery through competition, targets essential health services to specific population groups such as low income populations, pregnant women, children or the elderly, and acts as a means to change behavior [4,5]. OBA aims to cushion households from the catastrophic household expenditure associated with relatively high-cost interventions such as emergency obstetric care. The key elements include redeemable vouchers, health funds or insurance schemes that are intended to subsidize the target health services so that the out-of-pocket cost of medical care at the point of delivery becomes lower than the market price [5]. Demand side approaches seek to widen financial access and contribute to meeting Millennium Development Goal (MDG-5) which calls for significant reductions in maternal mortality [6].

To address the challenges of accessing reproductive health (RH) services, the Kenyan government with support from the German Development Bank (KfW) developed an OBA voucher program to subsidize selected RH services. Although a previous description has been documented [7], it focused on the initial phase of the program (2006–2008) and did not provide rigorous analysis of the implementation experiences. This paper explores how to work with the private and public sector using a voucher model, policy issues of targeting/benefiting populations and administrative complexities of implementing such a program [8]. We review the implementation experiences of the OBA program in Kenya with an aim of identifying lessons that can be used to provide policy directions in the implementation of similar programs in different settings and to scale. To do this we trace the implementation process of the Kenyan OBA program since inception (2006–2010) using policy analysis tools.

## Methods

There is an increasing recognition of the role of policy analysis in public health evaluation [9]. In addition, health system interventions have unpredictable paths of implementation and that interpretative, time-dependent decisions by different actors underpin the subsequent implementation process. We utilize the policy analysis framework, which emphasizes the need to take account of *who* (actors) and *how* (process) decisions are made, *what* (content) decisions are made and under *what* conditions (context) [10]. In addition, we examine the role of actors and their influence as a central theme through a stakeholder analysis [11,12] to draw out programmatic lessons for scale up.

This paper draws from two sets of data. First, a document review was conducted of available project and evaluation reports, publications and other relevant documents on the voucher project. From this we generated evidence on the dynamics of implementation, activities conducted and the decisions made over time. Documentary materials included seven design reports and contractual documents, five annual and midterm review reports, eight advisory and 20 steering committee minutes including four back-stopping mission reports.

The second set of data was qualitative interviews collected as part of the evaluation activities of the OBA programme in Kenya [13]. The overall aim of the qualitative component was to gain a deeper understanding of the perceptions of the actors on the programme. This paper draws from in-depth interviews (IDIs) conducted across five program sites: Kitui, Kiambu and Kisumu districts as well as Korogocho and Viwandani slums in Nairobi. Ten IDIs were conducted with health facility in-charges and 18 with service providers from the contracted facilities, District Medical Officers, Public Health Officers, local leaders and field managers.

In each site, a team of trained researchers conducted interviews with a standardized guide. Discussions with contracted providers, facility in-charges and field managers focused on their perceptions of programme design including accreditation, reimbursements, referral mechanisms, voucher distribution and perceived barriers to programme implementation. Interviews with local leaders examined access to reproductive health services and general community perceptions about the services, awareness of the programme, perceived impact and barriers to use of the vouchers at the community level.

Where consent was given qualitative interviews were recorded translated into English, transcribed and typed into Microsoft Word software. Debriefing sessions were held by the research team after each interview to provide an overview of issues raised. Informal analysis was conducted and summaries of the collected data made after each session for clarification or follow up. The data were stored and managed using QSR Nvivo8 Software (© QSR international Pty 2007, Australia).

Analysis of qualitative data entailed categorisation of issues based on inductive and deductive approaches by which *a priori* themes were used as a starting point. Later the thematic framework was improved as more data were examined [14]. Regular consultations were held with other members of the research team to enhance reflexivity. The analysis was also enriched by useful insights from members of the research team especially CW who was involved in the inception phase of the pilot program. Their views were useful in the interpretation on the role of actors and their influence on the implementation experiences.

Themes generated were further compared against analysis charts, which were developed based on the policy analysis framework [10]. Analysis charts were compared within and across sites to look for similarities and differences of key issues around implementation processes. Final analysis was organised around a description of the implementation process, role of actors, and power dynamics. A range of analyses examined experience within and across sites, with a view to identify complex interactions between key explanatory factors that account for the implementation practices at both national and district levels. We further drew from a complementary body of work on how to investigate power [15] to generate evidence on the importance of power dynamics on implementation of programs.

Ethical approvals were granted from Population Council Institutional Review Board and the Kenya Medical Research Institute (KEMRI) Ethics and Research Committee. Written informed consent were obtained from all the interviewees. To protect the identity of participants at the point of data collection and reporting is an important ethical procedure. However, a dilemma recognised in this study is lack of complete anonymity of data especially during reporting given the small number of actors being interviewed. Attempts were made to minimise these problems and strike a balance between the value of providing information on implementation experiences and anonymising participants. Interviewees were also given the options of not using voice recorders during interviews and to omit their quotes in reports and papers. Another measure used to maintain anonymity in reporting was the use of broad actor groups to indicate the perspective of the information without linking to a particular actor. This was important as certain information was considered sensitive but necessary to illustrate challenges of implementation.

## Results

### Program inception

The overarching goal of the OBA program is to improve access to RH services, decrease maternal and child deaths and increase acceptance of the long term family planning (FP) services. Document review suggested that this was to be achieved through provision of safe motherhood (SM), FP and gender-based violence recovery services (GBVRS) vouchers. The SM and FP vouchers targeted poor women while the GBVRS was to cater for all survivors regardless of socio-economic status. It was envisaged that the OBA would provide crucial experiences in targeting, accreditation, claims, reimbursement and quality for the then proposed National Social Health Insurance Fund. The program was designed in 2006 in view of Kenya’s poor maternal mortality indicators.

The planning process took several years from conception to program launch. Initial consultations were done late 2003 culminating in the creation of a technical support mission in early 2004 bringing together partners from government, donors, non-governmental organizations (NGOs) and faith-based organizations (FBOs) in a workshop where the voucher concept was introduced and discussed [16]. The results of the 2004 technical mission were presented in a feasibility report to the donors and the executing agency, the then National Coordinating Agency for Population and Development (NCAPD) renamed as the National Council for Population and Development in 2012. This included recommendations for the design, cost, and organizational structure of the program, investments in information systems, financial systems, capacity development and marketing strategies [17]. Following this, in early 2005, an agreement was reached between the governments of Kenya and Germany through the German Development Bank to fund RH services in Kenya through a voucher program with an estimated budget of 6.5 million Euros. These funds supported a pilot implementation (“Phase 1”) carried out over three years (2006–2008).

### Organizational arrangements and role of actors

**Figure 1:**
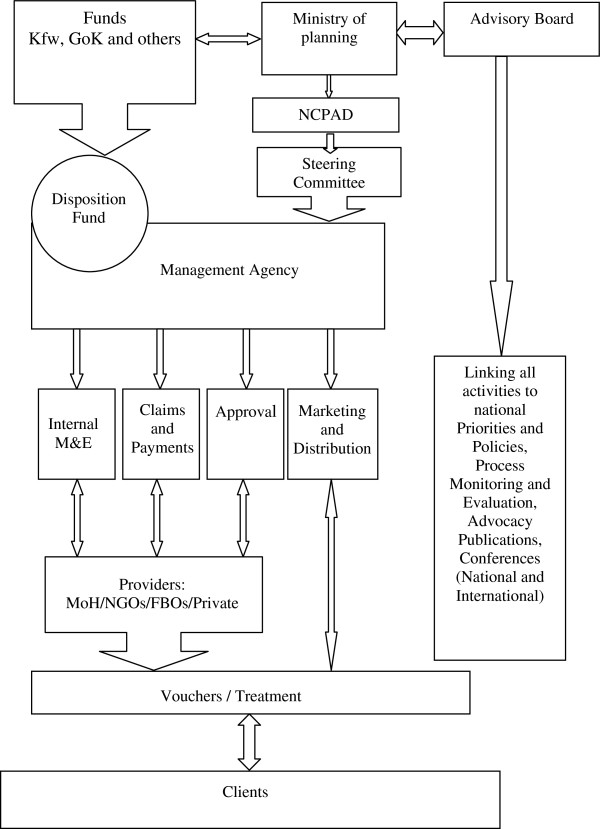
Organizational arrangement of the project.

The voucher program was established under the Ministry of Planning through NCAPD, mandated to oversee the implementation (Figure 1). NCAPD chaired essential organs of implementation and was instrumental in making decisions on design and management including contracting the voucher management agency. NCAPD being the executing agency was a powerful actor drawing their influence from their mandate (Table 1). The executive team was chaired by an enthusiastic team leader who was viewed as the champion of the OBA concept in Kenya and was responsible for popularising it within government circles. Qualitative interviews showed that this provided an opportunity for effective leadership and an avenue for donors and other government agencies to support the concept leading to effective implementation in the initial phases. Table 2 summarizes the role of actors involved and their influence during the implementation period.

**Table 1:** Force field map illustrating level of power and influence by actors involved

**Level of power**	**Proponents**			**Non mobilised**	** Opponents**		
	**High support**	**Middle**	**Low**		**Low**	**Middle**	**High opposition**
High	NCAPD						
	Advisory board						
	Steering Committee						
Medium	Price water house Coopers(PwC) - VMA	MoH-DRH					
		NHIF					
		IGES					
		Voucher distributors					
		Service Providers					
		Nairobi Women’s hospital					
Low		Marie Stopes	Microd Consult	Kalzmat Security			
		Population Council	Lowe Scanad	Kenya Medical educational Trust			

**Table 2:** Actors’ interests, position and influences on implementation process

**Category of Actors**	**Roles in the program**	**Interest**	**Level of power**	**Position**
The Project Executing Agency-NCAPD	The MoH was engaged in conceiving and planning the introduction NSHIF. For this reason, the MoH’s capacities to introduce new concepts and approaches into the service delivery system were stretched to the limit. Ministry of Planning and National Development offered to step in as the temporary host with the Programme Executing Agency being the NCAPD	High	High	Supportive
Advisory board	Provided support and advice on the medical, administrative, economic, and ethical matters. Promoted and maintained communication with the steering committee. Endorsed the VMA’s quarterly progress report and plans. Oversaw the OBA program implementation and link program to policy and political stream	High	High	Supportive
Steering committee	Reviewed and approved draft contracts for distributors and providers, including the fee schedule; all planed activities, quarterly budgets and operational plans, evaluation and audit reports. Monitoring and coordination of the program on behalf of GOK; provided backstopping where necessary and made decisions regarding the implementation, adaptation and improvement of the program	High	High	Supportive
MoH-DRH	Overall policy guidance with less involvement in the implementation	High	Medium	Supportive
Pricewaterhousecoopers Water House (PwC) VMA	Contracted to train VSP; developed and implemented a marketing and distribution strategy for the vouchers, Collection and processing of claims**;** paid for the services according to the terms of the contracts	High	Medium	Supportive
NHIF	Selected as the government agency mandated to conduct quality assurance. Accreditation and quality assurance	High	Medium	Middle support
Microd Consult	Selected after tendering to support NCPAD to develop a monitoring and evaluation framework but did not to play an active role	Low	Low	Middle support
Lowe Scanad	Marketing the program in various project sites	Low	Low	Middle support
**International actors**				
Marketing Consultant	Provided independent evaluation of the effectiveness of the marketing and distribution functions	Medium	Medium	Middle support
KfW	Provided funding and were instrumental in designing the concept	High	High	Supportive
Institut für Gesundheits- und Sozialforschung (IGES)	Selected as consultants to the programme during its starting phase and to periodically provide professional inputs on the concept, overall programme management, medical aspects associated with the different services covered by the vouchers, marketing and reimbursement systems. Mediating different stakeholders. Contracted to offer back stopping services since it was the only one with knowledge on the voucher program	High	Medium	Supportive
**Local actors**				
Voucher distributors	Marketing of the vouchers, interviewing clients to ensure target beneficiaries obtain the voucher	High	Medium	Supportive
Service providers	Providing services to voucher clients, prepare the requisite documentation on services provided for claims processing and report to VMA any irregularities noted on vouchers presented by clients	High	Medium	Supportive
Nairobi Women’s Hospital	Identified based on being the only facility that provided gender-based violence recovery services	High	Medium	Supportive
Kalzmat Security Print Ltd	Printing vouchers	Middle	Low	Immobilised
Marie Stopes	Developed and provision of a participatory poverty grading tool that was used to identify eligible clients for the voucher system	High	Low	Supportive
Kenya Medical and Educational Trust	Involved in initial design phases but not in implementation	Low	Low	Immobilised
Population Council	Involved in development of the accreditation criteria and standards for voucher package and the quality assurance manual	High	Low	Supportive
African Population and Health Research Center	Involved in initial design phases but not in implementation	Low	Low	Immobilized
**Community level actors**				
Beneficiaries	Responsible for seeking the vouchers and using them to access services at the accredited VSP’s. Required to provide accurate and true information to the voucher distributor which is used to determine their eligibility	High	Low	Supportive

The operation of the program was realized through two committees representing distinct interest groups. The committees carried out their activities within the NCAPD mandate. The first committee was the advisory board with an oversight role and its members drawn from NGOs, Ministry of Health (MoH), the German Society for International Cooperation (then GTZ, now GIZ), FBOs, and the private doctors’ association. The board held regular meetings to receive quarterly reports from the steering committee and make decisions on program functioning. It was chaired by the executive team of NCAPD and was highly influential in the planning, design, and implementation process at all levels. The second committee was the OBA Steering Committee (OSC) comprising representatives from NCAPD, MoH Department of Reproductive Health (DRH), KfW and the technical backstopping team. They met quarterly and defined operational procedures and organized meeting schedules. Although the committee reported to the advisory committee, their terms of reference provided them with ability to give direction on the program and make recommendations to the advisory committee on changes to be made.

The overall implementation process was managed by the voucher management agency (VMA)—a consortium of PricewaterhouseCoopers (PwC) and Population Council selected through a competitive selection process in October 2005. The Population Council’s role was limited and had little input in the project beyond the first year. Although Population Council contributed to the design phase, its role in the program can be viewed as a missed opportunity as their potential (given its expertise in reproductive health) was not utilized maximally leading to the council’s middle support for the program (Table 1).

The VMA reported directly to NCAPD through the Steering Committee. It was involved in the adaptation of the program to local settings. Although the VMA was criticized for not having public health expertise being a financial audit firm, their strength lay in its own internal management system that facilitated the implementation process including an internal monitoring and evaluation department, and claims processing section. They maintained direct contractual arrangements with the providers. The VMA can be viewed to have less “power” in relation to the NCAPD but in essence it had extensive influence amongst service providers, distributors and clients as indicated in Table 1. The VMA played a significant role during the initial phases of the program helping to generate evidence for scale up. The VMA, however, being the main implementer was not represented in the steering committee giving them little opportunity to directly discuss implementation issues and get resolutions to program-related problems in a timely manner.

Other actors include the MoH/DRH, which was less influential in the initial phase although the RH service delivery was in their docket. This may have resulted in missed opportunities as they did not provide the needed RH technical expertise leading to their middle position they occupied in terms of supporting the program (Table 1). The VMA also contracted Lowe Scanad, a local marketing firm to design a marketing strategy to create demand. In addition, a marketing consultant was hired for independent evaluation of the effectiveness of the voucher marketing and distribution functions. However, these actors contributed minimally over the program period resulting in low influence towards program implementation and are represented as actors with low support or were not well mobilized for effective implementation in Table 1.

The National Hospital Insurance Fund (NHIF) was given a contract to accredit voucher service providers (VSPs). This was made for technical as well as strategic reasons to gather political support from the government. It was selected to ensure continuity with the program in its capacity as the agency that conducts Standards and Quality Assurance of health service providers in the country. NHIF’s interests were in building more evidence on the potential for expanding universal access for medical insurance coverage. However, initial progress of the quality assurance process was not comprehensive enough; in particular, a lack of periodic system of monitoring quality led to an inability to ensure quality structures were put in place, which may have implied that NHIF lacked the resources and will to conduct quality assurance assessments.

### Analyzing the content and design of the program

Document review indicated that when the program was initiated it offered limited number of services but additional services were included once the concept of OBA was well understood amongst key actors. For example, the initial benefit package did not include antenatal care (ANC) services since its uptake was generally high but it was introduced later after extensive deliberations. The SM voucher thus covered ANC care, labor and delivery, caesarean section, postnatal care up to six weeks, as well as complications of pregnancy and childbirth at a subsidized price of $ 2.5^a^ to poor women (Table 3). Providers were reimbursed $ 44 for SM vouchers redeemed and an average of $ 225 for caesarian section deliveries. The FP voucher covered: intra uterine contraceptive devices (IUCD), implants, and surgical contraception (tubal ligation and vasectomy) at a cost of $ 1.25 with a reimbursement to providers of about $ 12 for non surgical procedures and $34 for surgical procedures. The GBVRS voucher was initially supposed to operate along the same lines as SM and FP, with specified limits for counseling and medical treatment by specific accredited service providers. However, due to the unpredictability of the event and the stigma it carries it was decided that the vouchers would only be available at the health facilities for free and be reimbursed at full cost of service.

**Table 3:** Key design features

**Key features**	**Characteristics**
**1. Benefit package**	
*Safe motherhood (SM)*	
Management of labour and complications	✓ Delivery- vacuum extraction and caesarean care
	✓ Emergency obstetric care; manage all stages of labour
	✓ Refer for emergency obstetric care services
	✓ Management of postpartum hemorrhage, eclampsia but not abortion related issues
	✓ Management of retained placenta, prolonged labour/obstructed, ruptured uterus.
	✓ Four ANC visits
Post natal care	✓ Within six hours of delivery examination of clients for danger signs
	✓ Referral for postpartum hemorrhage, third degree tear
	✓ Newborn care and referral for sick new born
	✓ Post operative care for Caesarean section
	✓ Review mother and counsel on infant feeding practices
	✓ Contraception and PMTCT services
Long term Family planning methods	✓ Long-term contraception methods but at present underutilized- implants, IUCD, male and female voluntary surgical contraception
Gender based violence	✓ Medical examination and treatment and management of injuries
	✓ Hospitalization and accommodation
	✓ Laboratory testing and X-rays including (HIV/AIDS, High Vaginal Swab, Hepatitis, Pregnancy, Syphilis, Urinalysis, Haemogram, Liver Function
	✓ Access to pregnancy prevention medication & antiretroviral drugs
	✓ Professional counselling
**2. Quality Assurance**	
Infrastructure and basic services	✓ Facilities with basic equipment and infrastructure and staff according to the level of care
	✓ Provision of basic emergency and comprehensive obstetric care
Monitoring quality	✓ Documentation of treatment given including filling in the partograph, patient file notes and register
	✓ Documentation to facilitate reimbursements and claims
	✓ Analysis of claims data regularly every three months
**3. Selection of facilities and accreditation process**	
Selection of providers	✓ Mapping of facilities in selected districts based on level of care and service given (basic and comprehensive obstetric care) and licensed service providers
	✓ Selection made by an assessor using set criteria
Contracting providers and distributors	✓ Contractual agreements with selected VSPs and distributors
**4. Marketing and distribution**	
Distribution process	✓ Two mechanisms used; direct approach distribution where vouchers are sold to clients in their homes; use of specific locations- where clients will go to specific points to access the vouchers such as preferred fixed selling points such as pharmacies.
Marketing strategy	✓ Designed to use local media, radio shows, vans buses, fliers and posters and marketed as VOCHA brand
**5. Claims and reimbursement process**	
Claims processing	✓ Time line pegged at a month from presenting claim with proper documentation
Reimbursement	✓ Reimbursement fee set based on a market analysis of what different facilities charged and negotiated
**6. Management system**	✓ System of managing program with clearly defined roles

### Contracting and quality assurance

Accreditation process was characterized by the development of criteria that was developed by Population Council and DRH. The criteria for accrediting FP and SM services were adapted from the existing national standards. There were no criteria that could be drawn for GBVRS; these were developed based on experience and local circumstances. NHIF was involved in selecting the VSPs and accredit them in consultation with the Technical Committee on Accreditation and Quality Assurance.

Data from the qualitative interviews show that accreditation of health facilities was adapted to local settings. During phase two some facilities were contracted even though they did not meet minimum standards with the aim of supporting competition and patient choice, and with the understanding that their service quality would improve over time: “*I think it’s the situation of the facility since it’s too interior and we also needed support since we are getting mothers delivering mostly with the Traditional Birth Attendants because they cannot afford delivering in the hospitals. And we also needed to improve the services in the facility” (Clinical Officer-Dispensary).* The aim was to nurture capacity and experience among providers. This was hoped that it would yield benefits beyond the scope of the voucher program. However, this resulted in limited services as some of the providers were unable to offer the full benefit package.

NHIF accredited a total of 54 VSPs to offer RH services during phase one. However, it was evident that there were differences between the VMA and NHIF regarding the process and timing of quality assurance mechanisms. While the VMA perceived that it is was sufficient to examine service quality once a year, NHIF was convinced that the increasing number of voucher clients required frequent monitoring and training of hospital staff. Although quality assurance inspection by the NHIF was planned to be done every six months, this was not adequately implemented. In August 2008, the technical committee was reconstituted under the leadership of the Head of Division of Family Health to undertake a review on the quality assurance status on the accredited VSPs. It was also evident from technical committee findings that the accreditation process was not well synchronized and lacked feedback mechanism to the providers. Overall the review of quality assurance procedures reveal that basic RH policies, guidelines and standards were generally not well implemented while the VSPs had very little or no access to refresher trainings and RH skills update from MoH.

### Marketing and distribution of vouchers

The Kenya voucher program was designed to utilise the existing local administrative structures, community and opinion leaders to popularise it. This was envisaged to play a vital role in creating awareness among the target community. However, community level discussions showed that the process was not effectively implemented. There was evidence that in some sites, the administrative offices provided venues for fixed distribution points for voucher sales. The distribution process utilised a poverty grading tool for both SM and FP services except the gender violence recovery services, which were made available in health facilities for all who needed it. The marketing strategy was generally not intense during the initial implementation period despite contracting a marketing firm. Qualitative interviews show promotional activities were targeted in specific locations and the information was limited to less remote settings leading to poor uptake of vouchers. Initial plans were to use multiple marketing campaign strategies such as local radio advertisement, road shows, and educational strategies. Use of radio broadcast turned out to work well in Kisumu, but none was available in the Nairobi slum area*.* In Kiambu, the response to radio spots was received with an overwhelming majority and many people from as far as Nairobi were attracted to participate without necessarily being eligible. This high demand led to a discontinuation of the radio spots. On the other hand, it was noted that the role of the marketing agency was potentially limited by budgetary constraints, time and complex marketing strategies required. The role of the agency was thus reduced to undertaking a one-off activity with limited interactions across sites resulting in their low influence suggesting lost opportunity to maximise their potential.

**Figure 2:**
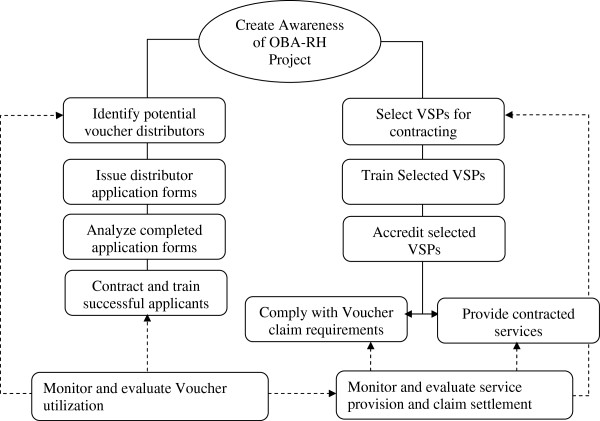
Voucher distribution process.

Voucher distribution was based on two approaches. One was a fixed point distribution linked to the commissioned agents who received 25% commission per voucher. This approach was not well executed leading to malpractices such as disregarding adherence to the poverty grading tool. In addition in some instances distributors attempted to sell vouchers to clients who were in extreme need such as in hospitals to women in early labour or in other instances selling to those who did not come from the target community or avoiding sparsely populated rural areas. Following this experience a decision was reached to hire full-time trained distributors on a monthly stipend. Utilising this approach had the unintended benefit of accommodating some women who could not afford to pay up front for vouchers as distributors were more likely to let women to pay in installments. In general though, there was a perception that there was need to further improve the distribution process. Figure 2 shows the arrangement behind the voucher distribution process.

### Claims processing and reimbursement

The claims process was one aspect that was elaborate and required adherence to procedure. Both interviews and documentary review showed that once clients visited an accredited facility with a voucher for services under the benefits package, the VSPs provided the service and then filed the necessary documentation for reimbursement. These documents include: the original voucher, duly completed service claim form, discharge summary or medical report, a copy of the patient’s or guardian’s identification card, and the original invoice and statement of account on the invoices being submitted to VMA. Submitted claims were first verified and then sent through the approval process. As soon as payments were ready they were wired into the VSP’s bank accounts and an advice sent to the VSP, advising on the particular payments effected and invoices included in the payment.

The program design provided for a reimbursement procedure that could process claims within 30 days through a computer system. However, this was not the case in practice as the process was perceived to be slow and cumbersome and did not account to actual payments made..: “*I think the process of reimbursement takes too long. There’s a time you claim and the time you are receiving this money it takes quite a while and you see when we send these claims; when they are reimbursing us they should also send a copy of what they have paid and what they have not paid explaining why what was not paid . ..... .... The last time I called there I was told there is a problem with the claim processing system; we should wait” (Provider).* Although sometimes the delay was occasioned by challenges of the claims system within the VMA, some facilities also violated the guidelines stipulated by attending to clients for conditions not included in the benefit package resulting in a claim rejection*.* In other cases claims were rejected because claim forms were signed by hospital staff on behalf of the clients, providers tampered with the voucher details or submitted incomplete, inconsistent or delayed documentation.

The VMA set ceilings for the reimbursement of various services based on an earlier study. The actual reimbursement rates were negotiated with each service provider based on the respective cost situation. Additional expenses from medical complications were covered by the VMA as long as they were deemed valid and documented well. In spite of this there were complaints around the ceiling set as providers in some private facilities suggested the amount was too little “ *........I also feel the amount of money they are giving us is not enough. For example if we have ANC; if our clients come here, the cash patients or non-OBA patient; they are usually 3 or 4 visits. For 3 visits, that is KES 900 and also the first visit when you come, we charge KES 400. Then there is the ANC profile done during the first ANC visit; our profile costs KES 1000. When you add this all up it is giving you around KES 2300 for a cash patient of which the OBA are giving us only KES 1000. When it comes to normal delivery, our normal delivery ranges from five to around eight or ten thousand but the OBA they are giving us four thousand. If it’s above four thousand, that is a complication. Come to Caesarean, our Caesarean ranges from about twenty-one to around thirty [thousand]. They are only giving us twenty thousand (Private provider).*

In some places, this resulted to voucher clients getting less attention than non-voucher clients who can pay higher prices. Moreover some private establishments were reluctant to participate although they were better located to serve target clients. Others who were accredited pulled out as a result of being overwhelmed by the demand created by voucher clients. Although, some providers felt obliged to treat all clients equally and not to discriminate against voucher clients, taking into account their capacity constraints, they could only do so by limiting access for voucher clients. This contradicted a major principle of the voucher systems that is client’s free choice among all accredited service providers.

Apart from processing claims and accessing the funds, providers working in public facilities reported challenges in utilizing the reimbursements from voucher clients. The bureaucratic barriers in the public health system meant that most public health facilities could not benefit from the proceeds of voucher clients. To address this there were long deliberations and consultations with the MoH and it was agreed that money generated from the OBA would be used by the public service providers to improve care (such as purchase of supplies, laboratory consumables, improve sanitary conditions) but not to hire additional staff on a permanent basis. Hiring staff on a temporarily basis was accepted. The general view among providers was that funds generated from voucher service delivery were to be exclusively used for improvement of quality service in reproductive health services; however, bureaucratic requirements limited its use; “*I am telling you now this money we are not able to use it as the OBA money. It is consolidated as the hospital money so trying to push it back to the facility like now the maternity it is a struggle. So we tried from the Ministry whether it can be banked as the OBA money and they refused. We cannot do that. It is against government policy. So if all this money was to be banked separately then we could be able to access the money 100% but now we are not able. Because now once the committee sits down to budget whatever we have collected it is included. And then the facility receives it as what they have budgeted for that facility,… although matron tries to insist that they should increase the allocation to the department” (Provider)*.

Overall, providers experienced challenges such as lack of awareness among clients and providers in the reimbursable services, delays in payment, lack of information on those claims that were rejected and inadequate communication on the procedures as one provider was quoted: “*feedback is not good at all , .... Like for example I told you we had a meeting in May and it’s just last month we received one payment. We don’t know for which batch the payment is because there was no advice slip that was sent. From then we have not received any other and yet we are still seeing these patients. They have not communicated back. We send a letter to them, nothing was said. We send another letter and it has not been replied” (Provider).*

### Implementation process

**Figure 3:**
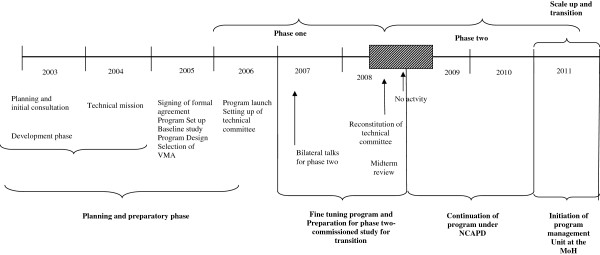
Time line of the implementation process.

The implementation process was designed in phases as shown in Figure 3. The phased implementation provided an opportunity for learning and adapting the program to local settings and making necessary changes. One important contextual event in Kenya that helped to drive the voucher agenda forward was the fact that parliament was considering adopting legislation to create a National Social Health Insurance Fund. The voucher concept was viewed by some Kenyan policymakers as a useful model to prepare for the envisaged national social health insurance fund.

Phase one was implemented between October 2005 and October 2008. The first nine months were characterized by planning and setting up of the program. The project was launched officially in the first pilot district in Kisumu in mid 2006 after a four month delay of project funds. This also delayed contracts for marketing, VSP accreditation and quality assurance. The project began with eight accredited health facilities in Kisumu district. By the end of 2006 facilities in two other pilot districts: Kiambu, Kitui and the slum areas in Nairobi were included. The second and third years (2007 and 2008) focused on fine tuning the program.

Phase II was preceded by several discussions leading to bilateral talks between the Kenya and German Governments in February 2007 and an allocation of about 10 million Euros. To facilitate smooth transition, scale up and institutionalization and sustainability of the project from NCAPD to the MoH, in May 2007, the steering committee agreed that the MoH would attach some of their staff to the project after the midterm review. Although a transition program was drawn after the midterm review, the realization of this phase took longer than anticipated. This was reflected by a period in 2008 where there were no activities undertaken during the transition period (refer to patterned phase of Figure 3). This was attributed to the long process which included a commissioned study to review the institutional framework and give recommendations on the possible transfer of the program from the NCAPD under the Ministry of State for Planning, National Development and Vision 2030 to the planning and finance department in the Ministry of Health.

The study recommendations noted the undergoing changes in the health sector and the future of the NHIF and the envisioned national social insurance fund were yet to be clearly defined. Thus it was recommended that the program activities be maintained under the management of NCAPD. A study was proposed to refine the technical design of phase II. The second phase was set to start in November 2008 and run to October 2011.

Later in 2008 a discussion paper was presented in a workshop proposing to include the OBA concept as a government mechanism. This provided a boost to the OBA concept within government leading to process of identifying it as flagship program for the vision 2030 [18]. During phase two, there were delays for a year in contracting the technical team, finally, a consulting firm (EPOS) was contracted to support the transition of the program to the MoH. This culminated to the development of a program management unit within the MoH to support the scale up.

### Program modifications

A number of changes were made by the Advisory Board over the implementation period most of which were based on ongoing learning. Firstly, there were transient contextual features. For example FP commodity stock outs, notably implants, resulted in low uptake and under utilization of long term FP services. Based on these experiences, the steering committee decided to use the process monitoring funds to support the DRH to ensure that no stock outs of implants were experienced over the implementation period. Secondly, during phase one, the government announced waivers for deliveries and maternity fees in government facilities—the announcement was made during an electioneering period. However, this did not affect the design directly, but necessitated more public information in the project sites. Thirdly, flexibility of the voucher programs meant mid-stream changes were possible. Through the support of German Government, a 6 million Euros food aid component was introduced. The food aid component was intended to meet the food requirements of the SM voucher clients and malnourished clients in need of gender based violence services. The World Food Program was contracted by KfW to implement the food aid component in liaison with the VSPs. The component was to run for 3 years. The rollout of the food aid component commenced in Nairobi and Kiambu district in July and August 2009 followed by Kitui and Kisumu districts in the subsequent months. In each of the sites, the proprietors of the health facilities and their staff were sensitized and trained on the food aid component before they were given the food for distribution. However, qualitative interviews suggest that this was misinterpreted in Kisumu, where beneficiaries of food supplements were associated with people living with HIV which generated stigma affecting uptake of FP vouchers.

There were changes in voucher distribution systems as lessons were learned. The VMA managed to regularly monitor the claims and delinked voucher distribution from VSP to avoid situations where providers and distributors could file claims for ghost clients. In some cases distributors sold vouchers at higher prices. The VMA responded by putting up posters on the prices of vouchers on market days. It was reported in some sites that wealthy patients bought the vouchers potentially crowding out the ability of the intended beneficiaries to access the voucher. In other places, some patients and VSPs copied the vouchers and there were incidents where providers refused to see voucher clients or charged extra amounts. The VMA conducted regular exit interviews and careful review of claims to control irregular behaviour and potential fraud.

In addition to changing management practices to deter fraud, the program added an family planning method in the first postnatal visit on the SM package and refined the scope of services for gender-based violence survivors. In addition, changes were made to the SM voucher, allowing an expectant mother to have several consultations -in line with standard 4 ANC visits- in the course of pregnancy and admissions during the last trimester (e.g. false labour). Prior to this change, providers had used copied vouchers to make a claim.

## Discussion

A systematic review on outcomes of RH voucher programs show that such strategies increase utilization of RH services, improve quality of care and population health outcomes. However, there is limited evidence on program effectiveness and cost-effectiveness as well as the analysis of implementation process of such programs to draw out lesson for scale up [19]. This paper attempts to fill this gap by providing systematic assessment of implementation process to provide lessons on how such programs can be implemented and scaled up.

Before illustrating lessons learnt for scale up, a number of limitations are worth highlighting. First, the analysis of implementation experiences is based on interviews and retrospective documentary analysis. Although the approach triangulated ideas from multiple sources, we were not able to corroborate all the views in reports. Finally, some members of the research team were involved in the inception phase of the voucher program and some element of reflexivity may have affected the research agenda including tools development and data analysis.

This study has identified a number of lessons from the implementation of a RH voucher program, drawing from three main areas: strategic management; implementation process and how design elements may have influenced implementation process and policy implications for scale up.

In terms of strategic management, three key lessons can be identified. First, use of an advisory board and a steering committee helped to institutionalize accountability, generated checks and balances, and allowed adaptation of program elements to local settings to improve implementation. The use of strategies such as pegging subsequent implementation activities on the success of the activities allowed for reflections of lessons learnt and provided an opportunity for modifications. This improved both implementation process and outputs. Although strategic decisions made during the deliberations of the regular committee meetings were communicated to the VMA, future management ought to consider including the VMA in the steering committee, perhaps as an observer, for timely implementation of the decisions made.

Secondly, in the early program, the NCAPD team leader was part of the steering committee that was influential, respected and enjoyed wide support from both government and donor community. In the second phase, the Director of Public Health and Sanitation had a critical role in decision-making on routine operational issues. Going forward, the program will be strengthened by further development of systems that are insulated from the routine turnover of key leaders. This institutionalization is underway with the development of a program management unit within the Ministry of Health.

Thirdly, strategic partnership between the private and public sector when well managed can help improve public health goods as was demonstrated by the involvement of PwC as the VMA. Contracting out is one feature that has been widely documented to facilitate effective delivery of health services but there is limited evidence of how this works in RH voucher programs [8]. This study shows that clear contractual arrangements need to be put in place to facilitate implementation of OBA programs.

The Kenyan experience has provided some insights on the dynamics of implementation of OBA programs. Detailed planning beforehand and use of feedback mechanisms allow for adjustment during the program. Good planning allows adequate time to account for unexpected challenges and having mechanisms to counter such situations through effective management strategies. This also allows smooth transition from the pilot phase to scale up. A similar assessment of the Bangladesh voucher program noted that careful planning was necessary before implementation to ensure that adequate administrative and financial resources are mobilized [5].

Implementation process will need to continue to optimize organizational learning and flexibility as experience is gained over time. This flexibility may be difficult in bureaucratic contexts, however, utilizing a private-public partnership as was the case for PwC may help to improve efficiency especially in service contracting and claims processing. Such arrangements need to ensure the objectives of the private sector and the public sector service delivery are aligned to produce high quality performance.

In terms of design changes as the program scales, there is a continual need to communicate the benefits package effectively to voucher service providers, including reminders of what information is reimbursable. This may necessitate periodic marketing using different media when the program goes to scale. Going forward, public sector facility autonomy could more clearly established. Policymakers should give the facility-level managers greater discretion on use of these reimbursement funds to improve quality of care for RH services or share across the facility. Another potential challenge that needs routine review at the planning and operational levels is the targeting the poor. The Kenyan experience illustrates that VMA can use local knowledge in addition to the poverty grading tool may improve targeting. However, further work needs to be done to provide evidence on targeting mechanisms.

The second aspect of design is the claims processing. For an effective system, an operational management information system ought to be considered to improve the voucher tracking system from point of service through claims review and reimbursement and allow for flexible addition of new information technology modules or services in the future. This would help improve payment speed and reduce fraud and error. The timely processing and disbursement of vouchers and incentive payments is likely to increase private sector participation and improve population-level access to maternal services.

## Conclusions

For effective scale up of RH voucher programs, such schemes will require an effective partnership between the public and private entities guided by clearly defined rules of engagement. The government should provide adequate funding, play a stewardship role and look for opportunities to utilize existing platforms to scale up such strategies. Active, cost-effective engagement with service providers in updating them and educating them on the process is likely to be a role that government should emphasize for effective participation generating wide buy-in. This paper has illustrated that RH voucher schemes can be implemented successfully as pilot program and has a potential for scaling up with stronger partnership between the public and private sectors.

## Endnote

^a^1$ = KES 89.

## Competing interests

The authors declare that they have no competing interests.

## Authors' contributions

TA was involved in the document review, analysis of data and was responsible for drafting the manuscript. CW, JO, RN, FO, and LK were involved in reviewing the manuscript for intellectual content. BB and IA were substantially involved in conceptualizing the study and revising the manuscript for intellectual content. All authors read and approved the final manuscript.

## Pre-publication history

The pre-publication history for this paper can be accessed here:

http://www.biomedcentral.com/1471-2458/12/540/prepub
